# HCV triple therapy in co-infection HIV/HCV is not associated with a different risk of developing major depressive disorder

**DOI:** 10.7448/IAS.17.4.19629

**Published:** 2014-11-02

**Authors:** Renata Fialho, Majella Keller, Alex File, Catherine Woods, Marco Pereira, Elaney Yousseff, Jeremy Tibble, Neil Harrison, Martin Fisher, Jennifer Rusted, Richard Whale

**Affiliations:** 1 University of Sussex and Sussex Partnership NHS Foundation Trust, Neuropharmacology Group, Brighton, UK; 2 Digestive Diseases Centre, Brighton and Sussex University Hospitals NHS Trust, Brighton, UK; 3 University of Coimbra, Cognitive and Behavioural Centre for Research and, Coimbra, Portugal; 4 Elton John Centre, Brighton and Sussex University Hospitals NHS Trust, Brighton, UK; 5 Brighton and Sussex Medical School, Brighton, UK; 6 School of Psychology, University of Sussex, Brighton, UK; 7 Neuropharmacology Group, Brighton and Sussex Medical School and Sussex Partnership NHS Foundation Trust, Brighton, UK

## Abstract

**Introduction:**

Hepatitis C (HCV) treatment options have changed with the development of direct activity antivirals (DAAs) and the availability of triple therapies have improved HCV cure rates. A common neuropsychiatric side effect of pegylated-interferon and ribavirin treatment is major depressive disorder (MDD), however little is known about such adverse events with protease inhibitor-based triple therapy. The aim of this study was to assess the rate of MDD in co-infected HIV HCV patients undergoing different HCV treatments.

**Methods:**

All participants were co-infected HIV HCV attending the Royal Sussex County Hospital Brighton hepatology outpatient clinic between 2010 and 2014. Participants were assessed for DSM-IV MDD and depression severity (using the Hamilton depression scale (HAMD)) at baseline and monthly after treatment initiation. HIV and HCV stages, genotype, reinfection and standard demographic variables were recorded. Influence of HCV stage (acute vs. chronic) and type of treatment (classic vs triple), emergence of MDD and clearance outcomes were analyzed using repeated measures and logistic regression models.

**Results:**

Fifty participants with a mean age of 42.65 years (SD=10.32) were included; most were male (98%). The majority had contracted HCV genotype 1 (64%) or 4 (26%). The HCV stage and treatment groups were matched for age and depression at baseline. No significant differences were found on virological outcomes considering HCV stage and treatment. From baseline to SVR, there was a significant increase in HAMD scores, F(4,36)=10.09, p<.001; this was not significantly influenced by HCV stage, F(4,35)=0.54, p=.708 or HCV treatment group, F(4,35)=0.60, p=.664. Those with chronic HCV were more likely to transition to MDD than acute infection (OR 7.77, 95% CI 2.04–29.54, p=.003). No differences were found for depression emergence by HCV treatment group (OR 0.83, 95% CI 0.22–3.13, p=.787).

**Conclusions:**

HCV triple therapy was not associated with a different risk of emergence of MDD versus classic treatment. MDD should be assessed before therapy initiation and monitored throughout treatment for any HCV treatment regime. Future research could usefully clarify mechanisms of MDD emergence and risk factors for this.

